# Supplementary material for: Tead1a Initiates Transcriptional Priming Through the TEAD1a/YAP‐Notch1‐Spi1/Cebpα Axis to Promote Neutrophil Fate
Source: Adv Sci (Weinh). 2025 Aug 28;12(41):e05441. doi: 10.1002/advs.202505441 (PMC12591127; doi:10.1002/advs.202505441)
Supplement: Supplementary file 4 — Supplementary Dataset [file ADVS-12-e05441-s004.zip › 3-1.zip!/3-1/02-1.cluster/cluster2/Enrich/ego_CC.pdf]

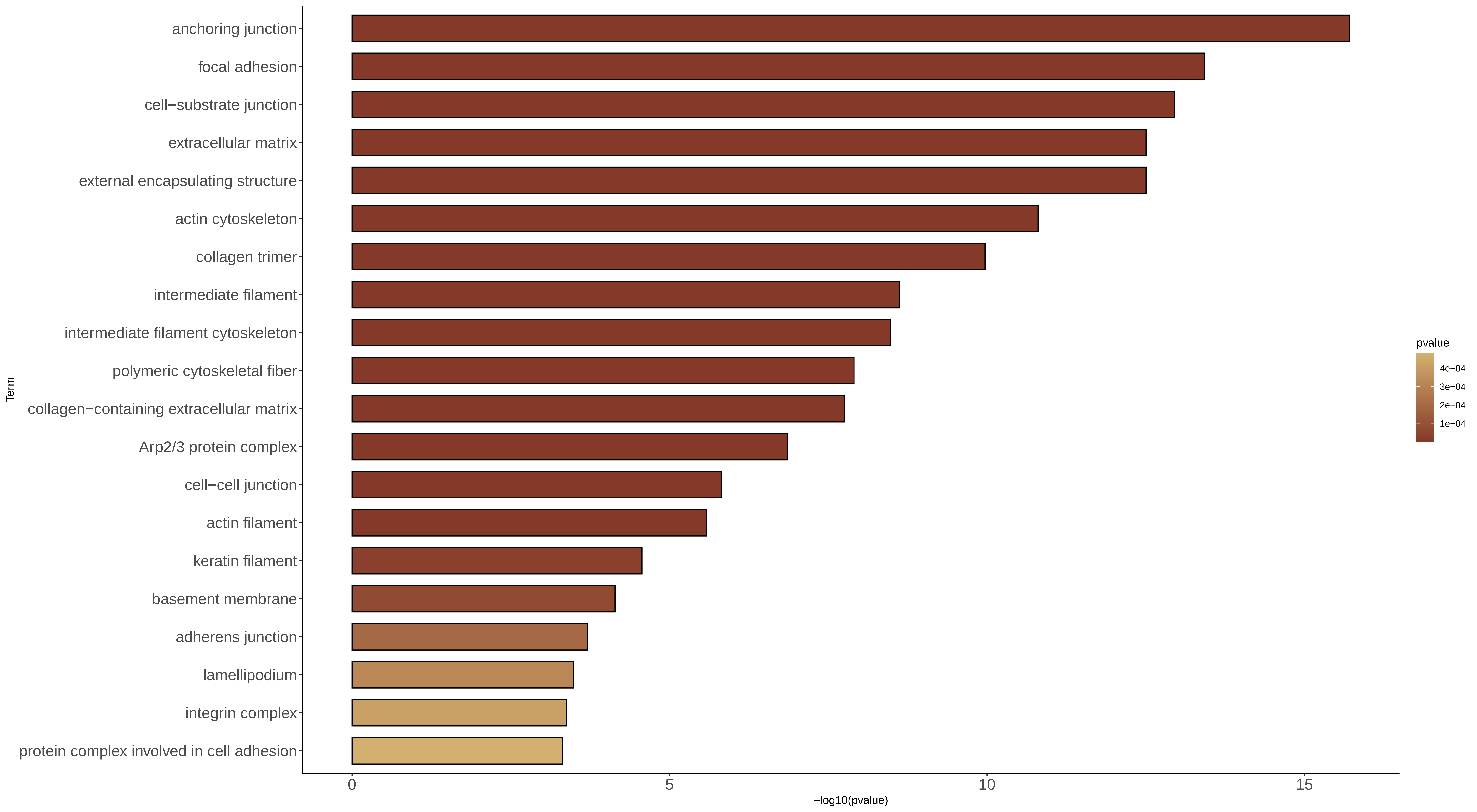
anchoring junction
focal adhesion
cell−substrate junction
extracellular matrix
external encapsulating structure
actin cytoskeleton
collagen trimer
intermediate filament
intermediate filament cytoskeleton
polymeric cytoskeletal fiber
collagen−containing extracellular matrix
Arp2/3 protein complex
cell−cell junction
actin filament
keratin filament
basement membrane
adherens junction
lamellipodium
integrin complex
protein complex involved in cell adhesion
Term
0
5
10
15
−log10(pvalue)
pvalue
4e−04
3e−04
2e−04
1e−04
